# Effects of COVID-19 on College Students’ Mental Health in the United States: Interview Survey Study

**DOI:** 10.2196/21279

**Published:** 2020-09-03

**Authors:** Changwon Son, Sudeep Hegde, Alec Smith, Xiaomei Wang, Farzan Sasangohar

**Affiliations:** 1 Department of Industrial and Systems Engineering Texas A&M University College Station, TX United States; 2 Center for Outcomes Research Houston Methodist Hospital Houston, TX United States

**Keywords:** COVID-19, pandemic, college student, mental health, stress, anxiety, self-management

## Abstract

**Background:**

Student mental health in higher education has been an increasing concern. The COVID-19 pandemic situation has brought this vulnerable population into renewed focus.

**Objective:**

Our study aims to conduct a timely assessment of the effects of the COVID-19 pandemic on the mental health of college students.

**Methods:**

We conducted interview surveys with 195 students at a large public university in the United States to understand the effects of the pandemic on their mental health and well-being. The data were analyzed through quantitative and qualitative methods.

**Results:**

Of the 195 students, 138 (71%) indicated increased stress and anxiety due to the COVID-19 outbreak. Multiple stressors were identified that contributed to the increased levels of stress, anxiety, and depressive thoughts among students. These included fear and worry about their own health and of their loved ones (177/195, 91% reported negative impacts of the pandemic), difficulty in concentrating (173/195, 89%), disruptions to sleeping patterns (168/195, 86%), decreased social interactions due to physical distancing (167/195, 86%), and increased concerns on academic performance (159/195, 82%). To cope with stress and anxiety, participants have sought support from others and helped themselves by adopting either negative or positive coping mechanisms.

**Conclusions:**

Due to the long-lasting pandemic situation and onerous measures such as lockdown and stay-at-home orders, the COVID-19 pandemic brings negative impacts on higher education. The findings of our study highlight the urgent need to develop interventions and preventive strategies to address the mental health of college students.

## Introduction

Mental health issues are the leading impediment to academic success. Mental illness can affect students’ motivation, concentration, and social interactions—crucial factors for students to succeed in higher education [1]. The 2019 Annual Report of the Center for Collegiate Mental Health [2] reported that anxiety continues to be the most common problem (62.7% of 82,685 respondents) among students who completed the Counseling Center Assessment of Psychological Symptoms, with clinicians also reporting that anxiety continues to be the most common diagnosis of the students that seek services at university counseling centers. Consistent with the national trend, Texas A&M University has seen a rise in the number of students seeking services for anxiety disorders over the past 8 years. In 2018, slightly over 50% of students reported anxiety as the main reason for seeking services. Despite the increasing need for mental health care services at postsecondary institutions, alarmingly, only a small portion of students committing suicide contact their institution counseling centers [3], perhaps due to the stigma associated with mental health. Such negative stigma surrounding mental health diagnosis and care has been found to correlate with a reduction in adherence to treatment and even early termination of treatment [4].

The COVID-19 pandemic has brought into focus the mental health of various affected populations. It is known that the prevalence of epidemics accentuates or creates new stressors including fear and worry for oneself or loved ones, constraints on physical movement and social activities due to quarantine, and sudden and radical lifestyle changes. A recent review of virus outbreaks and pandemics documented stressors such as infection fears, frustration, boredom, inadequate supplies, inadequate information, financial loss, and stigma [5]. Much of the current literature on psychological impacts of COVID-19 has emerged from the earliest hot spots in China. Although several studies have assessed mental health issues during epidemics, most have focused on health workers, patients, children, and the general population [6,7]. For example, a recent poll by The Kaiser Family Foundation showed that 47% of those sheltering in place reported negative mental health effects resulting from worry or stress related to COVID-19 [8]. Nelson et al [9] have found elevated levels of anxiety and depressive symptoms among general population samples in North America and Europe. However, with the exception of a few studies, notably from China [10-12], there is sparse evidence of the psychological or mental health effects of the current pandemic on college students, who are known to be a vulnerable population [13]. Although the findings from these studies thus far converge on the uptick of mental health issues among college students, the contributing factors may not necessarily be generalizable to populations in other countries. As highlighted in multiple recent correspondences, there is an urgent need to assess effects of the current pandemic on the mental health and well-being of college students [14-17].

The aim of this study is to identify major stressors associated with the COVID-19 pandemic and to understand their effects on college students’ mental health. This paper documents the findings from online interview surveys conducted in a large university system in Texas.

## Methods

### Study Design

A semistructured interview survey guide was designed with the purpose of assessing the mental health status of college students both quantitatively and qualitatively. In addition, the interview aimed to capture the ways that students have been coping with the stress associated with the pandemic situation. First, our study assesses participants’ general stress levels using the Perceived Stress Scale-10 (PSS) [18]. PSS is a widely used instrument to measure overall stress in the past month [19]. Second, participants were asked if their own and peers’ (two separate questions) stress and anxiety increased, decreased, or remained the same because of the COVID-19 pandemic. For those who indicated increased stress and anxiety during the pandemic, we questioned their stress coping strategies and use of available mental health counseling services. We then elicited pandemic-specific stressors and their manifestations across 12 academic-, health-, and lifestyle-related categories of outcomes such as effects on own or loved ones’ health, sleeping habits, eating habits, financial situation, changes to their living environment, academic workload, and social relations. Students were also asked about the impact of COVID-19 on depressive and suicidal thoughts. These constructs were derived from existing literature identifying prominent factors affecting college students’ mental health [20,21]. Feedback on the severity of COVID-19’s impact on these aspects were elicited using a 4-point scale: 0 (none), 1 (mild), 2 (moderate), and 3 (severe). Participants were asked to elaborate on each response. Third, participants were guided to describe stressors, coping strategies, and barriers to mental health treatment during a typical semester without associating with the COVID-19 pandemic. Although multiple analyses of the collected data are currently under progress, PSS results and the COVID-19–related findings are presented in this paper.

### Participants

**Figure 1:**
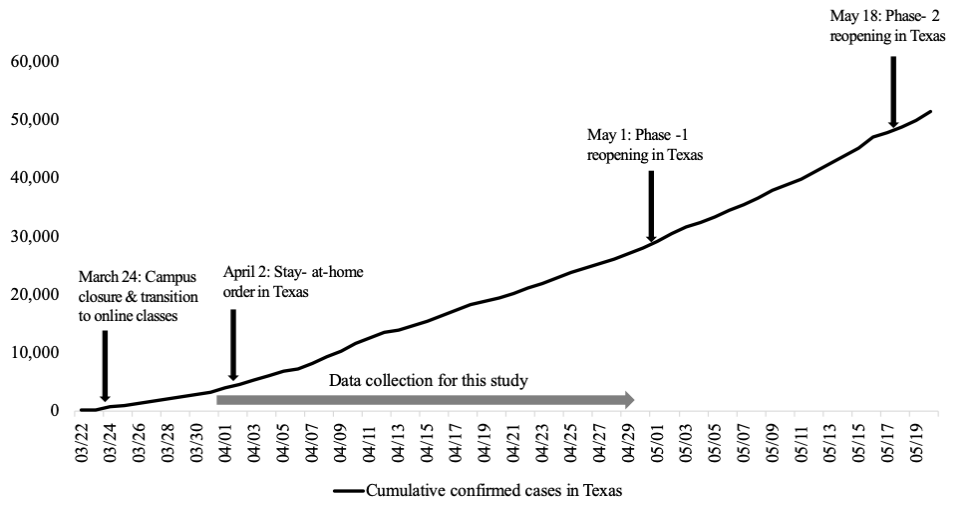
A timeline of major events related to COVID-19 in the university and the state of Texas (source: Texas Department of State Health Services).

Participants were recruited from the student population of a large university system in Texas, United States. This particular university closed all their campuses on March 23, 2020, and held all its classes virtually in response to the COVID-19 pandemic. In addition, the state of Texas issued a stay-at-home order on April 2, 2020. Most interviews were conducted about 1 month after the stay-at-home order in April 2020. Figure 1 illustrates the trend of cumulative confirmed cases and a timeline of major events that took place in the university and the state of Texas. Participants were recruited by undergraduate student researchers through email, text messaging, and snowball sampling. The only inclusion criteria for participation was that participants should have been enrolled as undergraduate students in the university at the time of the interviews.

### Procedures

The interviews were conducted by 20 undergraduate researchers trained in qualitative methods and the use of the interview survey guide described above. None of the authors conducted the interviews. All interviews were conducted via Zoom [22] and were audio recorded. The recordings were later transcribed using Otter.ai [23], an artificial intelligence–based transcription service, and verified for accuracy manually. Prior to the interview, participants were provided an information document about the study approved by the university’s Institutional Review Board (No 2019-1341D). Upon verbal consent, participants were asked to respond to a questionnaire about their demographic information such as age, gender, year of college, and program of study before completing the interview. Participation was voluntary and participants were not compensated.

### Data Analysis

First, descriptive statistics were compiled to describe participants’ demographics (eg, age, gender, academic year, and major) and the distribution of the ratings on PSS-10 survey items. A total PSS score per participant was calculated by first reversing the scores of the positive items (4-7, 9, and 10) and then adding all the ten scores. A mean (SD) PSS score was computed to evaluate the overall level of stress and anxiety among the participants during the COVID-19 pandemic. Second, participants’ answers to 12 academic-, health-, and lifestyle-related questions were analyzed to understand relative impacts of the pandemic on various aspects of college students’ mental health. Percentages of participants who indicated negative ratings (ie, mild, moderate, or severe influence) on these questions were calculated and ranked in a descending order. Qualitative answers to the 12 stressors and coping strategies were analyzed using thematic analysis [24,25] similar to the deductive coding step in the grounded theory method [26]. A single coder (CS), trained in qualitative analysis methods, analyzed the transcripts and identified themes using an open coding process, which does not use a priori codes or codes created prior to the analysis and places an emphasis on information that can be extracted directly from the data. Following the identification of themes, the coder discussed the codes with two other coders (XW and AS) trained in qualitative analysis and mental health research to resolve discrepancies among related themes and discuss saturation. The coders consisted of two Ph.D. students and one postdoctoral fellow at the same university. MAXQDA (VERBI GmbH) [27] was used as a computer software program to carry out the qualitative analysis.

## Results

### Participants

Of the 266 university students initially recruited by the undergraduate researchers, 17 retreated and 249 participated in this study. There were 3 graduate students and 51 participants who had missing data points and were excluded, and data from 195 participants were used in the analysis. The average age was 20.7 (SD 1.7) years, and there were more female students (111/195, 57%) than male students (84/195, 43%). Approximately 70% of the participants were junior and senior students. About 60% of the participants were majoring in the college of engineering, which was the largest college in the university population (Table 1). The mean PSS score for the 195 participants was 18.8 (SD 4.9), indicating moderate perceived stress in the month prior to the interview (Table 2).

**Table 1:** Participants’ demographic characteristics.

Variables		Participants (N=195)
Age (years), mean (SD)		20.7 (1.7)
**Gender, n (%)**		
	Male	84 (43.1)
	Female	111 (56.9)
**Academic year, n (%)**		
	Freshmen	24 (12.3)
	Sophomore	33 (16.9)
	Junior	70 (35.9)
	Senior	68 (34.9)
**Major (college), n (%)**		
	Agriculture & life science	10 (5.1)
	Engineering	117 (60.0)
	Liberal arts	20 (10.3)
	Architecture	1 (0.5)
	Business management	11 (5.6)
	Education and human development	12 (6.1)
	School of public health	5 (2.5)
	Science	5 (2.5)
	Veterinary medicine and biomedical sciences	10 (5.1)
	Not specified	4 (2.1)

**Table 2:** Mean score for each of PSS items.

PSS^a^ items	Score, mean (SD)
1. In the past month, how often have you felt upset because of something that happened unexpectedly?	2.2 (0.9)
2. In the past month, how often have you felt that you were unable to control the important things in your life?	2.2 (1.0)
3. In the past month, how often have you felt nervous and “stressed”?	2.8 (0.9)
4. In the past month, how often have you dealt successfully with irritating life hassles?	1.5 (0.9)
5. In the past month, how often have you felt that you were effectively coping with important changes that were occurring in your life?	1.5 (0.9)
6. In the past month, how often have you felt confident about your ability to handle your personal problems?	1.3 (0.9)
7. In the past month, how often have you felt that things were going your way?	1.9 (0.8)
8. In the past month, how often have you found that you could not cope with all the things that you needed to do?	1.8 (1.0)
9. In the past month, how often have you been able to control irritations in your life?	1.5 (0.9)
10. In the past month, how often have you felt that you were on top of things?	1.9 (1.0)
Overall PSS scores	18.8 (4.9)

^a^PSS: Perceived Stress Scale-10.

### Challenges to College Students’ Mental Health During COVID-19

**Figure 2:**
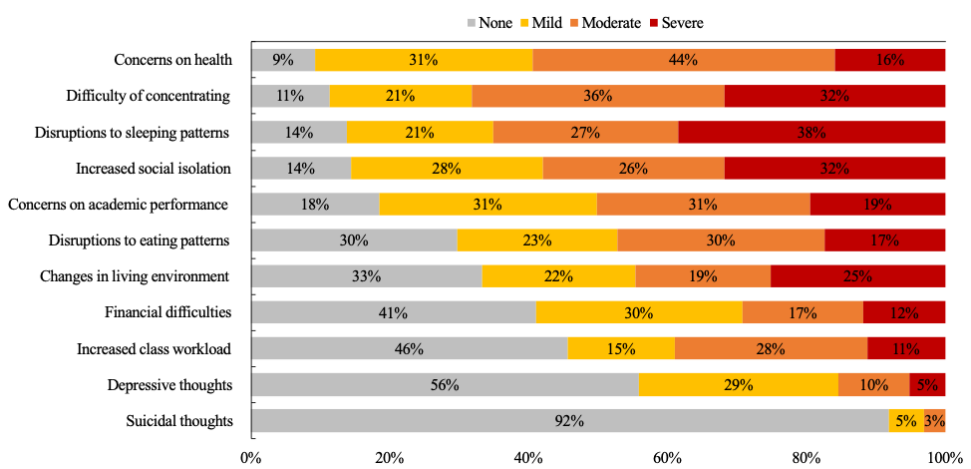
Participants’ ratings on mental health aspects in an order of negative impacts (mild, moderate, and severe).

Out of 195 participants, 138 (71%) indicated that their stress and anxiety had increased due to the COVID-19 pandemic, whereas 39 (20%) indicated it remained the same and 18 (9%) mentioned that the stress and anxiety had actually decreased. Among those who perceived increased stress and anxiety, only 10 (5%) used mental health counseling services. A vast majority of the participants (n=189, 97%) presumed that other students were experiencing similar stress and anxiety because of COVID-19. As shown in Figure 2, at least 54% (up to 91% for some categories) of participants indicated negative impacts (either mild, moderate, or severe) of COVID-19 on academic-, health-, and lifestyle-related outcomes. The qualitative analysis yielded two to five themes for each category of outcomes. The chronic health conditions category was excluded from the qualitative analysis due to insufficient qualitative response. Table 3 presents the description and frequency of the themes and select participant quotes.

**Table 3:** Categories and themes of college students’ mental health issues and selected participant quotes.

Theme		Participants^a^, n (%)	Example quotes^b^
**Own health and the health of loved ones (n=177)**			
	Worry about families and relatives with higher vulnerabilities	76 (43)	JPP17: “I have a grandma who is affected more by [the virus] and so I'm just afraid that something could happen to her.” SAP16: “My sister just had a baby on Friday and so I've just been worried that her baby or she wouldn't get anything.”
	Worry about families with more interpersonal contact	26 (15)	JJP06: “My brother just graduated from a med school and he is doing residency. So, every single patient he sees right now is most likely related to COVID-19.” DDP01: “My mom is actually an essential worker. She works at [a company] warehouse in [a city in Texas]. So, she's coming into contact with people every day.”
	Worry about themselves being infected	19 (11)	ACP05: “I always end up having runny nose or just asthma flares up. With this pandemic, the symptoms are very similar to corona so I feel like I would be one of those people who would be highly affected by it.”
**Difficulty in concentration (n=173)**			
	Home as a source of distraction	79 (46)	EGP03: “I'm having difficulty concentrating since I'm home. As I'm around all of my family, it's really hard to focus on what I need to do.”
	Lack of accountability and motivation	21 (12)	SAP16: “I just want to lay in my bed. Now no one is keeping me accountable. If I'm on my phone, I'm not paying attention to any of these lectures.”
	Distracted by social media, internet, and video games	19 (11)	DDP01: “My desk is right next to my bed so I could just go take a nap or go watch Netflix. Or I could just be on Twitter the whole time and read all the news stories about how people are dying or how bad this is going to get.”
	Lack of interactive learning environment	18 (10)	SAP17: “I cannot focus on class when it’s online. Through the classes, I don't think there's a lot of interactiveness to make people engaged.”
	Monotony of life	5 (3)	DDP07: “Now I'm stuck only doing everything on a computer. So, I'm pretty much on the computer all day.”
**Sleeping habits (n=168)**			
	Stay up later or waking up later	84 (50)	DDP03: “I'll be up until probably four or five in the morning, and sleep through the day usually. Now that most of my classes are online and the lecture isn't mandatory, I sleep through it and I'll watch the lectures later.”
	Irregular sleep patterns	28 (17)	SAP03: “I had a really weird sleep schedule now. I stay up really late. And then I wake up very early or sometimes I go to sleep early. I wake up really late. It is just weird.”
	Increased hours of sleep	12 (7)	ACP06: “I’m sleeping a lot more now. I’m living at home. I don’t have to do anything. I just have more time to sleep.”
	Difficulty of going/staying asleep	10 (6)	DDP07: “Now I wake up constantly. I wake up and go to sleep constantly. I have a hard time staying asleep and going asleep.”
**Social relation/social isolation (n=167)**			
	Reduced interactions with people	91 (54)	MBP01: “We're in quarantine so there is significant social isolation from people and from those that I want to hang out with.”
	Lack of in-person interactions	52 (31)	JJP02: “I don’t see my friends that much and no face to face interaction but only through text.”
	Restricted outdoor activities	9 (5)	SNK10: “I also like meeting new people so sometimes I go out climbing or hiking. [COVID-19] has impacted me a lot. I'm not able to do that anymore.”
**Academic performance (n=159)**			
	Challenges of online classes	61 (38)	RMP10: “It's so hard to focus on the lecture because everything is online. And I have to make appointments with a professor or a TA^c^. Then they help me through the Zoom which is online. I think it's hard to have some understanding compared to the face to face meeting.”
	Impacts on academic progress and future career	36 (23)	ACP07: “The class I wanted to take over the summer has been canceled, which could potentially push me back a semester.” RMP17: “I think my internship is going to be shortened or cancelled. I need to get more work experience before graduation.”
	Worry about grades	23 (14)	ACP12: “Shortly after COVID-19 was declared a pandemic, everything went online. We missed a week of class. So, I had four exams back to back but I didn't transition to online very well. I failed three out of four exams pretty badly. That also got me questioning my entire life and my major.”
	Reduced motivation or procrastination	12 (8)	RMP12: “I feel like I started slacking. I was trying to avoid this situation by just not doing some of the work. So, it is stressful academically.”
**Eating patterns (n=137)**			
	Increased eating/snacking	35 (26)	SNK08: “I've been munching a lot on snacks recently since I'm at home.”
	Inconsistent eating	27 (20)	SAP02: “I'm home all the time. Sometimes I eat twice a day. Sometimes I don't eat at all. Sometimes it's once a day. It's not something I haven't done before.”
	Decreased appetite	16 (12)	SAP15: “I'm having trouble eating. I just don't eat when I'm anxious. So, I've had no appetite.”
	Emotional eating	7 (5)	SAP04: “I eat so much now just out of boredom because there's nothing to do really.”
**Changes in living environment (n=130)**			
	Changes while staying back home	89 (68)	YJP05: “I moved back home. So, things are different here. I am having to study now in my bedroom rather than in the library or on campus.” JJP07: “By living with family, you don’t have any privacy. You don’t feel very focused because you are distracted.”
	Reduced personal interactions	18 (14)	ACP02: “I live in the dorm and everybody is moving out so there’s basically nobody around me anymore.”
	Staying longer indoor	9 (7)	RMP19: “Now I'm at home. I'm literally sitting in the same desk for five or six hours a day.”
**Financial difficulties (n=115)**			
	Impacts on current or future employment	44 (38)	SAP13: “I have rent to pay in [a local town] and I am not sure about my internship this summer. So, I'm going to be basically even in more debt and not unable to pay my bills and my rent.”
	Impacts on financial situations of families	21 (18)	ERP03: “My mom has so much that she needs to pay on her own. And she got deduction on her payment, but she still need to pay the same thing. She needs to pay for housing from both mine and my brothers, which is a lot.”
**Class workload (n=106)**			
	Catching up with online courses and class projects	51 (48)	ERP04: “[Professors] still want me to go to a Zoom class. Some of them still record those Zoom meetings and then you can watch it on your own time. It basically doubles the time I have to dedicate each week for that class.”
	Increased or more difficult assignments	33 (31)	ERP02: “Four or five out of my six professors have given more work than I would have had if I was there in person. Some of them have to do with participation, just proving that you actually watch the lecture or take notes for the class.”
	Difficulty of covering the same coursework in shorter time	6 (6)	NEP04: “A two-week break because of the pandemic made us compress that lost time into our last time we had scheduled.”
**Depressive thoughts (n=86)**			
	Loneliness	28 (33)	MBP02: “I actually suffer from chronic depression. [COVID-19] has definitely made it a lot worse, just being in isolation and being home 24/7. It feels like I need to get out but there's nowhere to go.”
	Insecurity or uncertainty	10 (12)	RMP18: “The first couple of days, it was very scary and I think everybody just felt like the world is ending.”
	Powerlessness or hopelessness	9 (10)	SNK01: “Maybe [COVID-19] made me really down. Sometimes I feel like I'm incompetent.” SAP20: “It's very easy to fall into a routine of nothingness. And you're seeing no end to this. It's just hopelessness about going back to normal.”
	Concerns about academic performance	7 (8)	ACP07: “A lot of hackathons I wanted to go to and a lot of research conferences I wanted to go to have all been shut down. And now it feels like all the work I have been doing for the last few months has been thrown away into the garbage.”
	Overthinking	4 (5)	SAP08: “There's just a lot and also you start going crazy in your apartment.”
**Suicidal thoughts (n=16)**			
	Linking to depressive thoughts	6 (38)	JJP03: “[Suicidal thoughts] go hand in hand with depressive thoughts. I am just tired of existing because I am just too hard on myself.” ECP02: “It just has to do with the depressive thoughts and just overthinking. You have a lot of time to think about things that happened in the past like high school. But there's no fixing it. Now, I'm stuck.”
	Academic issues	1 (6)	ACP12: “I hate to say it but it comes up on a daily basis. Sometimes as a joke, I want to die. But it's something that I know I have no intention to ever act on and never would like. It's just become incorporated in my life purposely or unconsciously when I do something especially related to academics.”
	Problems with parents	1 (6)	SNK09: “I have some problems with my family. And now I'm stuck at home with them. I guess it's more often than normal.”
	Fear from insecurity	1 (6)	JPP18: “The biggest thing has been fear of what's next. I think the worst part is more fear of what is to come and what will be the outcome.”

^a^Not every participant provided sufficient elaboration to allow for identification of themes, so the frequency of individual themes does not add up to the total number of participants who indicated negative impacts of the COVID-19 outbreak.

^b^The five-digit alphanumeric value indicates the participant ID.

^c^TA: teaching assistant.

#### Concerns for One’s Own Health and the Health of Loved Ones

A vast majority of the participants (177/195, 91%) indicated that COVID-19 increased the level of fear and worry about their own health and the health of their loved ones. Over one-third of those who showed concern (76/177, 43%) were worried about their families and relatives who were more vulnerable, such as older adults, those with existing health problems, and those who are pregnant or gave birth to a child recently. Some of the participants (26/177, 15%) expressed their worry about their family members whose occupation increased their risk of exposure to COVID-19 such as essential and health care workers. Some participants (19/177, 11%) specifically mentioned that they were worried about contracting the virus.

#### Difficulty With Concentration

A vast majority of participants (173/195, 89%) indicated difficulty in concentrating on academic work due to various sources of distraction. Nearly half of them (79/173, 46%) mentioned that their home is a distractive environment and a more suitable place to relax rather than to study. Participants mentioned that they were more prone to be interrupted by their family members and household chores at home. Other factors affecting students’ concentration were lack of accountability (21/173, 12%) and social media, internet, and video games (19/173, 11%). Some (18/173, 10%) stated that online classes were subject to distraction due to lack of interactions and prolonged attention to a computer screen. Additionally, monotonous life patterns were mentioned by some to negatively affect concentration on academic work (5/173, 3%).

#### Disruption to Sleep Patterns

A majority of participants (168/195, 86%) reported disruptions to their sleep patterns caused by the COVID-19 pandemic, with over one-third (38%) reporting such disruptions as severe. Half of students who reported some disruption (84/168, 50%) stated that they tended to stay up later or wake up later than they did before the COVID-19 outbreak. Another disruptive impact brought by the pandemic was irregular sleep patterns such as inconsistent time to go to bed and to wake up from day to day (28/168, 17%). Some (12/168, 7%) reported increased hours of sleep, while others (10/168, 6%) had poor sleep quality.

#### Increased Social Isolation

A majority of participants answered that the pandemic has increased the level of social isolation (167/195, 86%). Over half of these students (91/167, 54%) indicated that their overall interactions with other people such as friends had decreased significantly. In particular, about one-third (52/167, 31%) shared their worries about a lack of in-person interactions such as face-to-face meetings. Others (9/167, 5%) stated that disruptions to their outdoor activities (eg, jogging, hiking) have affected their mental health.

#### Concerns About Academic Performance

A majority of participants (159/195, 82%) showed concerns about their academic performance being impacted by the pandemic. The biggest perceived challenge was the transition to online classes (61/159, 38%). In particular, participants stated their concerns about sudden changes in the syllabus, the quality of the classes, technical issues with online applications, and the difficulty of learning online. Many participants (36/159, 23%) were worried about progress in research and class projects because of restrictions put in place to keep social distancing and the lack of physical interactions with other students. Some participants (23/159, 14%) mentioned the uncertainty about their grades under the online learning environment to be a major stressor. Others (12/159, 8%) indicated their reduced motivation to learn and tendency to procrastinate.

#### Disruptions to Eating Patterns

COVID-19 has also negatively impacted a large portion of participants’ dietary patterns (137/195, 70%). Many (35/137, 26%) stated that the amount of eating has increased, including having more snacks since healthy dietary options were reduced, and others (27/137, 20%) addressed that their eating patterns have become inconsistent because of COVID-19, for example, irregular times of eating and skipping meals. Some students (16/137, 12%) reported decreased appetite, whereas others (7/137, 5%) were experiencing emotional eating or a tendency to eat when bored. On the other hand, some students (28/195, 14%) reported that they were having healthier diets, as they were cooking at home and not eating out as much as they used to.

#### Changes in the Living Environment

A large portion of the participants (130/195, 67%) described that the pandemic has resulted in significant changes in their living conditions. A majority of these students (89/130, 68%) referred to living with family members as being less independent and the environment to be more distractive. For those who stayed in their residence either on- or off-campus (18/130, 14%), a main change in their living environment was reduced personal interactions with roommates. Some (9/130, 7%) mentioned that staying inside longer due to self-quarantine or shelter-in-place orders was a primary change in their living circumstances.

#### Financial Difficulties

More than half of the participants (115/195, 59%) expressed their concerns about their financial situations being impacted by COVID-19. Many (44/115, 38%) noted that COVID-19 has impacted or is likely to impact their own current and future employment opportunities such as part-time jobs and internships. Some (21/115, 18%) revealed the financial difficulties of their family members, mostly parents, getting laid off or receiving pay cuts in the wake of COVID-19.

#### Increased Class Workload

The effect of COVID-19 on class workload among the college students was not conclusive. Although slightly over half of participants (106/195, 54%) indicated their academic workload has increased due to COVID-19, the rest stated the workload has remained the same (70/195, 36%) or rather decreased (19/195, 10%). For those who were experiencing increased workloads, nearly half (51/106, 48%) thought they needed to increase their own efforts to catch up with online classes and class projects given the lack of in-person support from instructors or teaching assistants. About one-third of the participants (33/106, 31%) perceived that assignments had increased or became harder to do. Some (6/106, 6%) found that covering the remainder of coursework as the classes resumed after the 2-week break to be challenging.

#### Depressive Thoughts

When asked about the impact of the COVID-19 pandemic on depressive thoughts, 44% (86/195) mentioned that they were experiencing some depressive thoughts during the COVID-19 pandemic. Major contributors to such depressive thoughts were loneliness (28/86, 33%), insecurity or uncertainty (10/86, 12%), powerlessness or hopelessness (9/86, 10%), concerns about academic performance (7/86, 8%), and overthinking (4/86, 5%).

#### Suicidal Thoughts

Out of 195 participants, 16 (8%) stated that the pandemic has led to some suicidal thoughts with 5% (10/16) reporting these thoughts as mild and 3% (6/16) as moderate. There were 6 participants (38%) that attributed their suicidal thoughts to the presence of depressive thoughts. Other reasons were related to academic performance (1/16, 6%), problems with family as they returned home (1/16, 6%), and fear from insecurity and uncertainty (1/16, 6%).

### Coping Mechanism During COVID-19

To cope with stress and anxiety imposed by COVID-19, college students reported seeking support from others but were mainly using various self-management methods.

#### Self-Management

The majority of the participants (105/138, 76%) with increased stress due to the outbreak of COVID-19 explained that they were using various means to help themselves cope with stress and anxiety during the pandemic. Some (24/105, 23%) relied on negative coping methods such as ignoring the news about COVID-19 (10/105), sleeping longer (7/105), distracting themselves by doing other tasks (5/105), and drinking or smoking (2/105). Approximately one-third (30/105, 29%) used positive coping methods such as meditation and breathing exercises (18/105), spiritual measures (7/105), keeping routines (4/105), and positive reframing (2/105). A majority of the participants (73/105, 70%) who used self-management mentioned doing relaxing hobbies including physical exercise (31/105), enjoying streaming services and social media (22/105), playing with pets (7/105), journaling (5/105), listening to music (4/105), reading (2/105), and drawing (2/105). Finally, some participants (15/105, 14%) stated that they were planning activities (eg, drafting to-do lists) for academic work and personal matters as a self-distraction method.

#### Seeking Support From Others

Approximately one-third of the participants (47/138, 34%) mentioned that communicating with their families and friends was a primary way to deal with stress and anxiety during COVID-19. Some explicitly stated that they were using a virtual meeting application such as Zoom frequently to connect to friends and family. Only 1 participant claimed to be receiving support from a professional therapist, and another participant was using Sanvello, a mobile mental health service app provided by the university.

#### Barriers to Seeking Professional Support During COVID-19

Despite the availability of tele-counseling and widespread promotion of such services by the university, a vast majority of participants who indicated an increase in stress and anxiety (128/138, 93%) claimed that they had not used school counseling services during the pandemic. Reasons for such low use included the condition not being perceived as severe enough to seek the services (4/128, 3%), not comfortable interacting with unfamiliar people (1/128, 0.8%), not comfortable talking about mental health issues over the phone (1/128, 0.8%), and lack of trust in the counseling services (1/128, 0.8%).

## Discussion

### Principal Findings

College students comprise a population that is considered particularly vulnerable to mental health concerns. The findings of this study bring into focus the effects of pandemic-related transitions on the mental health and well-being of this specific population. Our findings suggest a considerable negative impact of the COVID-19 pandemic on a variety of academic-, health-, and lifestyle-related outcomes. By conducting online survey interviews in the midst of the pandemic, we found that a majority of the participants were experiencing increased stress and anxiety due to COVID-19. In addition, results of the PSS showed moderate levels of stress among our participants. This is in line with a recent pre–COVID-19 survey conducted in the United Kingdom (mean PSS score 19.79, SD 6.37) [28]; however, the administration of PSS as interview questions (compared to allowing participants to read and respond to the 10 questions) might have introduced bias and resulted in underreporting.

Among the effects of the pandemic identified, the most prominent was worries about one’s own health and the health of loved ones, followed by difficulty concentrating. These findings are in line with recent studies in China that also found concerns relating to health of oneself and of family members being highly prevalent among the general population during the pandemic. Difficulty in concentrating, frequently expressed by our participants, has previously been shown to adversely affect students’ confidence in themselves [29], which has known correlations to increased stress and mental health [30]. In comparison with stress and anxiety in college students’ general life, it appears that countermeasures put in place against COVID-19, such as shelter-in-place orders and social distancing practices, may have underpinned significant changes in students’ lives. For example, a vast majority of the participants noted changes in social relationships, largely due to limited physical interactions with their families and friends. This is similar to recent findings of deteriorated mental health status among Chinese students [10] and increased internet search queries on negative thoughts in the United States [31]. The findings on the impact of the pandemic on sleeping and eating habits are also a cause for concern, as these variables have known correlations with depressive symptoms and anxiety [20].

Although a majority of participants expressed concerns regarding academic performance, interestingly, almost half of the participants reported lower stress levels related to academic pressure and class workload since the pandemic began. This may be due, in part, to decisions taken by professors and the university to ease the students’ sudden transition to distance learning. For instance, this university allowed students to choose a pass/fail option for each course instead of a regular letter grade. Additionally, actions taken by professors, such as reduced course loads, open book examinations, and other allowances on grading requirements, could also have contributed to alleviating or reducing stress. Although participants who returned to their parental home reported concerns about distractions and independence, students might have benefited from family support and reduced social responsibilities. Therefore, the increased stress due to the pandemic may have been offset, at least to some extent.

Alarmingly, 44% (86/195) of the participants reported experiencing an increased level of depressive thoughts, and 8% (16/195) reported having suicidal thoughts associated with the COVID-19 pandemic. Previous research [32] reported about 3%-7% of the college student population to have suicidal thoughts outside of the pandemic situation. Furthermore, with the exception of high-burnout categories, depression levels among students, reported in several recent studies [33-35], have varied between 29% and 38%, which may suggest an uptick in pandemic-related depressive symptoms among college students similar to recent studies in China [10,11]. Although our participants specifically mentioned several factors such as feelings of loneliness, powerlessness, as well as financial and academic uncertainties, other outcomes that were perceived to be impacted by the COVID-19 pandemic may also act as contributors to depressive thoughts and suicidal ideation. In particular, both difficulty concentrating and changes in sleeping habits are associated with depression [20,29,36].

Our study also identifies several coping mechanisms varying between adaptive and maladaptive behaviors. The maladaptive coping behaviors such as denial and disengagement have been shown to be significant predictors of depression among young adults [37]. In contrast, adaptive coping such as acceptance and proactive behaviors are known to positively impact mental health. Our findings suggest that the majority of our participants exhibited maladaptive coping behaviors. Identifying students’ coping behavior is important to inform the planning and design of support systems. In this regard, participatory models of intervention development can be used, in which researchers’ and psychologists’ engagement with the target population to adapt interventional programs to their specific context has shown promise [37,38]. For instance, Nastasi et al [37] used a participatory model to develop culture-specific mental health services for high school students in Sri Lanka. Similar approaches can be adopted to engage college students as well to develop a mental health program that leverages their natural positive coping behaviors and addresses their specific challenges.

Participants described several barriers to seeking help, such as lack of trust in counseling services and low comfort levels in sharing mental health issues with others, which may be indicative of stigma. Perceiving social stigma as a barrier to seeking help and availing counseling services and other support is common among students [29]. One study showed that only a minor fraction of students who screened positive for a mental health problem actually sought help [39]. Although overcoming the stigma associated with mental health has been discussed at length, practical ways of mitigating this societal challenge remains a gap [40,41]. Our findings suggest that self-management is preferred by students and should be supported in future work. Digital technologies and telehealth applications have shown some promise to enable self-management of mental health issues [42]. For instance, Youn et al [43] successfully used social media networks as a means to reach out to college students and screen for depression by administering a standardized scale, the Patient Health Questionnaire-9. Digital web-based platforms have also been proposed to enhance awareness and communication with care providers to reduce stigma related to mental health among children in underserved communities [44]. For instance, one of the online modules suggested by the authors involves providing information on community-identified barriers to communicating with care providers. Technologies such as mobile apps and smart wearable sensors can also be leveraged to enable self-management and communication with caregivers.

In light of the aforementioned projections of continued COVID-19 cases at the time of this writing [45] and our findings, there is a need for immediate attention to and support for students and other vulnerable groups who have mental health issues [17]. As suggested by a recent study [46] based on the Italian experience of this pandemic, it is essential to assess the population’s stress levels and psychosocial adjustment to plan for necessary support mechanisms, especially during the recovery phase, as well as for similar events in the future. Although the COVID-19 pandemic seems to have resulted in a widespread forced adoption of telehealth services to deliver psychiatric and mental health support, more research is needed to investigate use beyond COVID-19 as well as to improve preparedness for rapid virtualization of psychiatric counseling or tele-psychiatry [47-49].

### Limitations and Future Work

To our knowledge, this is the first effort in documenting the psychological impacts of the COVID-19 pandemic on a representative sample of college students in the United States via a virtual interview survey method in the middle of the pandemic. However, several limitations should be noted. First, the sample size for our interview survey was relatively small compared to typical survey-only studies; however, the survey interview approach affords the capture of elaboration and additional clarifying details, and therefore complements the survey-based approaches of prior studies focusing on student mental health during this pandemic [10,11,50]. Second, the sample used is from one large university, and findings may not generalize to all college students. However, given the nationwide similarities in universities transitioning to virtual classes and similar stay-at-home orders, we expect reasonable generalizability of these findings. Additionally, a majority of our participants were from engineering majors. Therefore, future work is needed to use a stratified nationwide sample across wider disciplines to verify and amend these findings. Third, although a vast majority of participants answered that they have not used the university counseling service during the pandemic, only a few of them provided reasons. Since finding specific reasons behind the low use is a key to increasing college students’ uptake of available counseling support, future research is warranted to unveil underlying factors that hinder college students’ access to mental health support. Finally, we did not analyze how student mental health problems differ by demographic characteristics (eg, age, gender, academic year, major) or other personal and social contexts (eg, income, religion, use of substances).

Future work could focus on more deeply probing the relationships between various coping mechanisms and stressors. Additionally, further study is needed to determine the effects of the pandemic on students’ mental health and well-being in its later phases beyond the peak period. As seen in the case of health care workers in the aftermath of the severe acute respiratory syndrome outbreak, there is a possibility that the effects of the pandemic on students may linger for a period beyond the peak of the COVID-19 pandemic itself [51].

## Abbreviations

PSS: Perceived Stress Scale-10

## References

[1] Unger K (2007). Handbook on Supported Education: Providing Services for Students With Psychiatric Disabilities.

[2] (2020). 2019 annual report. Center for Collegiate Mental Health.

[3] Shuchman M (2007). Falling through the cracks — Virginia Tech and the restructuring of college mental health services. N Engl J Med.

[4] Eisenberg D, Downs MF, Golberstein E, Zivin K (2009). Stigma and help seeking for mental health among college students. Med Care Res Rev.

[5] Brooks SK, Webster RK, Smith LE, Woodland L, Wessely S, Greenberg N, Rubin GJ (2020). The psychological impact of quarantine and how to reduce it: rapid review of the evidence. Lancet.

[6] Lai J, Ma S, Wang Y, Cai Z, Hu J, Wei N, Wu J, Du H, Chen T, Li R, Tan H, Kang L, Yao L, Huang M, Wang H, Wang G, Liu Z, Hu S (2020). Factors associated with mental health outcomes among health care workers exposed to coronavirus disease 2019. JAMA Netw Open.

[7] Xie X, Xue Q, Zhou Y, Zhu K, Liu Q, Zhang J, Song R (2020). Mental health status among children in home confinement during the coronavirus disease 2019 outbreak in Hubei Province, China. JAMA Pediatr.

[8] Kirzinger A, Kearney A, Hamel L, Brodie M (2020). KFF health tracking poll - early April 2020: the impact Of coronavirus on life In America. Kaiser Family Foundation.

[9] Nelson B, Pettitt A, Flannery J, Allen N (2020). Rapid assessment of psychological and epidemiological correlates of COVID-19 concern, financial strain, and health-related behavior change in a large online sample. Int J Methods in Psychiatr Res.

[10] Cao W, Fang Z, Hou G, Han M, Xu X, Dong J, Zheng J (2020). The psychological impact of the COVID-19 epidemic on college students in China. Psychiatry Res.

[11] Liu X, Liu J, Zhong X (2020). Psychological state of college students during COVID-19 epidemic. SSRN J.

[12] Wang C, Zhao H (2020). The impact of COVID-19 on anxiety in Chinese university students. Front Psychol.

[13] Bruffaerts R, Mortier P, Kiekens G, Auerbach RP, Cuijpers P, Demyttenaere K, Green JG, Nock MK, Kessler RC (2018). Mental health problems in college freshmen: prevalence and academic functioning. J Affect Disord.

[14] Zhai Y, Du X (2020). Addressing collegiate mental health amid COVID-19 pandemic. Psychiatry Res.

[15] Zhai Y, Du X (2020). Mental health care for international Chinese students affected by the COVID-19 outbreak. Lancet Psychiatry.

[16] de Oliveira Araújo FJ, de Lima LSA, Cidade PIM, Nobre CB, Neto MLR (2020). Impact of Sars-Cov-2 and its reverberation in global higher education and mental health. Psychiatry Res.

[17] Holmes EA, O'Connor RC, Perry VH, Tracey I, Wessely S, Arseneault L, Ballard C, Christensen H, Cohen Silver R, Everall I, Ford T, John A, Kabir T, King K, Madan I, Michie S, Przybylski AK, Shafran R, Sweeney A, Worthman CM, Yardley L, Cowan K, Cope C, Hotopf M, Bullmore E (2020). Multidisciplinary research priorities for the COVID-19 pandemic: a call for action for mental health science. Lancet Psychiatry.

[18] Cohen S, Kamarck T, Mermelstein R (1983). A global measure of perceived stress. J Health Soc Behav.

[19] Cohen S, Kessler R, Gordon L (1997). Measuring stress: A Guide for Health and Social Scientists.

[20] Acharya L, Jin L, Collins W (2018). College life is stressful today - emerging stressors and depressive symptoms in college students. J Am Coll Health.

[21] Baghurst T, Kelley BC (2014). An examination of stress in college students over the course of a semester. Health Promot Pract.

[22] Zoom. Zoom Video Communications.

[23] Otter.ai. https://otter.ai/login.

[24] Braun V, Clarke V (2006). Using thematic analysis in psychology. Qualitative Res Psychol.

[25] Guest G, MacQueen K, Namey E (2011). Applied Thematic Analysis.

[26] Corbin J, Strauss A (2014). Basics of Qualitative Research (3rd ed.): Techniques and Procedures for Developing Grounded Theory.

[27] MAXQDA. VERBI Software.

[28] Denovan A, Dagnall N, Dhingra K, Grogan S (2017). Evaluating the perceived stress scale among UK university students: implications for stress measurement and management. Stud Higher Education.

[29] Martin JM (2010). Stigma and student mental health in higher education. Higher Education Res Dev.

[30] Zuckerman DM (1989). Stress, self-esteem, and mental health: how does gender make a difference?. Sex Roles.

[31] Jacobson NC, Lekkas D, Price G, Heinz MV, Song M, O'Malley AJ, Barr PJ (2020). Flattening the mental health curve: COVID-19 stay-at-home orders are associated with alterations in mental health search behavior in the United States. JMIR Ment Health.

[32] Zivin K, Eisenberg D, Gollust SE, Golberstein E (2009). Persistence of mental health problems and needs in a college student population. J Affect Disord.

[33] Zeng Y, Wang G, Xie C, Hu X, Reinhardt JD (2019). Prevalence and correlates of depression, anxiety and symptoms of stress in vocational college nursing students from Sichuan, China: a cross-sectional study. Psychol Health Med.

[34] Nahas ARMF, Elkalmi R, Al-Shami A, Elsayed T (2019). Prevalence of depression among health sciences students: findings from a public university in Malaysia. J Pharm Bioallied Sci.

[35] Fitzpatrick O, Biesma R, Conroy RM, McGarvey A (2019). Prevalence and relationship between burnout and depression in our future doctors: a cross-sectional study in a cohort of preclinical and clinical medical students in Ireland. BMJ Open.

[36] Heiligenstein E, Guenther G, Hsu K, Herman K (1996). Depression and academic impairment in college students. J Am Coll Health.

[37] Nastasi BK, Sarkar S, Varjas K, Jayasena A (1998). Participatory model of mental health programming: lessons learned from work in a developing country. Sch Psychol Rev.

[38] Cappella E, Jackson DR, Bilal C, Hamre BK, Soulé C (2011). Bridging mental health and education in urban elementary schools: participatory research to inform intervention development. Sch Psychol Rev.

[39] Eisenberg D, Hunt J, Speer N, Zivin K (2011). Mental health service utilization among college students in the United States. J Nerv Ment Dis.

[40] Husain W (2020). Barriers in seeking psychological help: public perception in Pakistan. Community Ment Health J.

[41] Fischer EP, McSweeney JC, Wright P, Cheney A, Curran GM, Henderson K, Fortney JC (2016). Overcoming barriers to sustained engagement in mental health care: perspectives of rural veterans and providers. J Rural Health.

[42] Torous J, Jän Myrick K, Rauseo-Ricupero N, Firth J (2020). Digital mental health and COVID-19: using technology today to accelerate the curve on access and quality tomorrow. JMIR Ment Health.

[43] Youn SJ, Trinh N, Shyu I, Chang T, Fava M, Kvedar J, Yeung A (2013). Using online social media, Facebook, in screening for major depressive disorder among college students. Int J Clin Health Psychol.

[44] Ginossar T, Nelson S (2010). Reducing the health and digital divides: a model for using community-based participatory research approach to e-health interventions in low-income Hispanic communities. J Computer-Mediated Commun.

[45] COVID-19 projections. Institute for Health Metrics and Evaluation.

[46] de Girolamo G, Cerveri G, Clerici M, Monzani E, Spinogatti F, Starace F, Tura G, Vita A (2020). Mental health in the coronavirus disease 2019 emergency-the Italian response. JAMA Psychiatry.

[47] Zhou J, Liu L, Xue P, Yang X, Tang X (2020). Mental health response to the COVID-19 outbreak in China. Am J Psychiatry.

[48] Zhou X, Snoswell CL, Harding LE, Bambling M, Edirippulige S, Bai X, Smith AC (2020). The role of telehealth in reducing the mental health burden from COVID-19. Telemed J E Health.

[49] Shore JH, Waugh M, Calderone J, Donahue A, Rodriguez J, Peters D, Thomas M, Giese A (2020). Evaluation of telepsychiatry-enabled perinatal integrated care. Psychiatr Serv.

[50] Huckins JF, daSilva AW, Wang W, Hedlund E, Rogers C, Nepal SK, Wu J, Obuchi M, Murphy EI, Meyer ML, Wagner DD, Holtzheimer PE, Campbell AT (2020). Mental health and behavior of college students during the early phases of the COVID-19 pandemic: longitudinal smartphone and ecological momentary assessment study. J Med Internet Res.

[51] McAlonan GM, Lee AM, Cheung V, Cheung C, Tsang KW, Sham PC, Chua SE, Wong JG (2007). Immediate and sustained psychological impact of an emerging infectious disease outbreak on health care workers. Can J Psychiatry.

